# Distinct Hemodynamic and Morphological Characteristics of Arteries Adjacent to White Matter Hyperintensities

**DOI:** 10.1111/cns.70673

**Published:** 2025-11-24

**Authors:** Boyu Zhang, Yan Han, Yajing Huo, Zidong Yang, Hongwei Li, Huihui Lv, Xiaotao Tai, He Wang

**Affiliations:** ^1^ Department of Radiology Shanghai Fourth People's Hospital Affliated to Tongji University School of Medicine Shanghai China; ^2^ Department of Neurology, Yueyang Hospital of Integrated Traditional Chinese and Western Medicine Shanghai University of Traditional Chinese Medicine Shanghai China; ^3^ USC Viterbi School of Engineering University of Southern California Los Angeles California USA; ^4^ Laboratory of FMRI Technology, USC Mark and Mary Stevens Neuroimaging and Informatics Institute, Keck School of Medicine University of Southern California Los Angeles California USA; ^5^ Institute of Science and Technology for Brain‐Inspired Intelligence Fudan University Shanghai China; ^6^ School of Second Clinical Medicine Yunnan University of Chinese Medicine Kunming China

**Keywords:** arteries, blood flow, blood pressure, cerebral small vessel diseases, white matter hyperintensity

## Abstract

**Objective:**

Altered cerebral perfusion has been implicated in the development of white matter hyperintensities (WMHs), yet the specific influence of hemodynamic features in proximal arteries on WMH burden remains uncertain. This study aimed to investigate the relationship between arterial flow characteristics and WMH severity.

**Methods:**

A total of 2631 subjects (68.6 ± 11.1 years, 50.3% female) who underwent MRI (magnetic resonance imaging) and MRA (magnetic resonance angiography) scans were involved in this retrospective observational study. Using an individualized simplified hemodynamic model, we derived arterial flow rate, mean pressure, and pressure drop for each MRA‐visible branch. WMHs were quantified on T2‐FLAIR images and categorized into periventricular and deep subtypes. The associations between arterial features and WMH burden were examined using general linear models.

**Results:**

Higher mean flow rate (*β* = 0.10, 95% CI: 0.06–0.14, *p* < 0.001) and mean pressure (*β* = 0.03, 95% CI: 0.02–0.04, *p* < 0.001) were associated with increased WMH volume. Adjacent‐to‐lesion terminal arterial branches (ALTAB), which represented arteries surrounding WMH, exhibited greater length (21.7 ± 8.21 mm vs. 13.3 ± 2.35 mm, *p* < 0.001), greater tortuosity (1.52 ± 0.39 vs. 1.26 ± 0.11, *p* < 0.001), lower mean flow rates (0.40 ± 0.09 mL/min vs. 0.90 ± 0.25 mL/min, *p* < 0.001) and lower pressure drops (0.42 ± 0.16 mmHg vs. 0.54 ± 0.15 mmHg, *p* < 0.001) compared to distant arteries. Greater WMH volume was found to be associated with an increased number of ALTAB.

**Conclusion:**

The hemodynamic features of arteries surrounding WMH exhibited significant differences compared to those located further away. Such changes in arterial morphology and corresponding hemodynamic features might be associated with the severity of WMH.

## Graphical abstract

In this retrospective, observational study involving 2631 individuals, cerebral arterial hemodynamic characteristics were associated with white matter hyperintensity. Moreover, the hemodynamic features of arteries surrounding WMH exhibited a significant difference compared to those located further away.

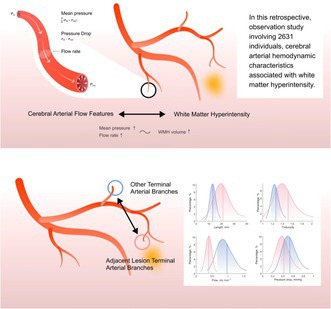

**Abbreviations**
ALTAB: adjacent‐to‐lesion terminal arterial branch
CSVD: cerebral small vessel disease
DWMH: deep white matter hyperintensity
PWMH: periventricular white matter hyperintensity
TOF: time‐of‐flight
WMH: white matter hyperintensity

## 1 Introduction

Cerebral small vessel disease (CSVD) is a pathological condition predominantly affecting the small penetrating arteries, arterioles, capillaries, and venules in the brain, leading to various physiological and cognitive abnormalities, including white matter hyperintensities (WMH) [1, 2]. WMH, characterized by white matter hyperintense regions on T2‐weighted MRI sequences, serves as a radiological hallmark of CSVD, presenting a significant neurobiological interest due to its prevalence in the elderly population and its association with cognitive decline, dementia, and stroke [3, 4]. Moreover, WMH has been implicated in increased risks of depression, impaired gait, and a higher risk for cerebrovascular disease, all of which significantly diminish the well‐being of individuals affected [5, 6, 7]. Understanding the intricacies of the risk for WMH would not only offer insights into the cerebrovascular diseases associated but also pave the path for potential therapeutic interventions to prevent relevant damage.

Multiple studies have advanced our understanding of the cerebrovascular basis of WMH. Autopsy investigations frequently reveal that WMHs are associated with small‐vessel pathologies such as arteriolosclerosis, lipohyalinosis, and venous collagenosis, together with myelin loss, gliosis, and axonal damage, implicating ischemia and blood–brain barrier dysfunction in WMH pathology [8, 9]. Perfusion MRI studies consistently show reduced cerebral blood flow in both WMH and normal‐appearing white matter in individuals with high WMH burden, and predict WMH progression over time [10, 11]. Meanwhile, the “vascular watershed hypothesis” posits that regions lying at borders between arterial supply territories, especially internal watershed zones in deep white matter, are particularly vulnerable to hypoperfusion and thus develop WMHs more readily [12, 13]. These findings collectively establish that WMHs are not merely incidental or age‐related MRI phenomena but reflect vascular injury, perfusion deficiency, and structural microvascular compromise.

As an organ with a profound demand for blood supply, the brain is highly sensitive to variations in blood flow [14]. Studies have highlighted that subtle alterations in cerebral blood flow could have significant implications for cognitive functions and overall neural health [15, 16, 17]. Therefore, monitoring and understanding the dynamics of blood flow in the brain is crucial in clinical settings. Traditionally, studies have employed sophisticated hemodynamic models for more accurate simulation of cerebral flow. Given the need for the input of precise boundary conditions and highly specialized requisites to deploy such models to estimate cerebral blood flow properly, those models are less capable of handling large‐scale clinical image data [18, 19]. The inherent nuanced complexities of hemodynamic models have limited their accessibility to a niche group of dedicated experts and veiled their translational potential in large‐scale clinical studies aimed at uncovering the relationship between cerebral hemodynamics and CSVD.

To investigate the complex nature of cerebral arterial hemodynamics and relations with WMH, the individual‐specific simplified hemodynamic model, a computational blood flow model that used certain assumptions (steady‐flow, patient‐specific arterial geometry) to estimate blood flow and pressure without requiring full fluid dynamics simulations, was employed in a large group of patients free of stroke [8, 9]. A cardinal focus is to identify the association of arterial flow features with WMH. Moreover, an evaluation of the difference in flow features between adjacent lesion terminal arteries and other terminal arteries was presented to improve the understanding of the influence of WMH lesions on surrounding arteries. WMHs were classified as the periventricular WMH (PWMH) or deep WMH (DWMH) to further study the differential effects of arterial flow features on anatomically distinctive lesions, and the WMH lesion‐based analysis was also reported.

## 2 Materials and Methods

### 2.1 Study Population

This retrospective, observational study was approved by the Ethics Committee of Yueyang Hospital of Integrated Traditional Chinese and Western Medicine, Shanghai University of Traditional Chinese Medicine in accordance with the Declaration of Helsinki (No. 2020‐060). The study used only previously collected data, with no additional tests or interventions undertaken on any participants and thus would inflict no impact on patient care or outcome. Therefore, the written informed patient consent was not included in this study. The inclusion criteria were: (1) hospitalized in the Department of Neurology between January 2017 and June 2021; (2) age greater than 18 years; and (3) complete cranial MRI and MRA. Patients with the following conditions were excluded: (1) secondary WMH, such as immunological, infectious, toxic, metabolic, and other causes; (2) abnormal brain lesions, such as brain trauma, intracerebral hemorrhage, non‐lacunar cerebral infarction, and other intracranial space‐occupying lesions; and (3) MR angiography showing severe intracerebral atherosclerotic stenosis (≥ 50%). As reported previously, the subjects involved were mostly elderly individuals with traditional cerebrovascular risk factors [10]. The main reasons for their visits were due to: headache, dizziness, sleep disorders, palpitations, gait disturbances, memory loss, or concentration problems. Patients with CT‐confirmed signs of CSVD were admitted for hospitalization, and conventional BP and heart rate monitoring and brain MRI were performed for further evaluation of CSVD.

The demographic and clinical characteristics, including age, sex, cigarette use, alcohol consumption, hypertension, diabetes, hyperlipidemia, history of cardiovascular disease, were collected from the clinical record. Hypertension was defined as self‐reported hypertension, treatment with antihypertensive agents, systolic BP ≥ 140 mmHg, or diastolic BP ≥ 90 mmHg.

### 2.2 Brain MRI and Analysis

Individuals underwent brain MRI and MRA scans on one 3.0T MR scanner (Philips Ingenia; Philips Healthcare, Best, the Netherlands). The MRI protocol included T1‐weighted structural imaging (TE = 2.3 ms, TR = 250 ms, flip angle = 75°, FOV = 230 mm × 230 mm, pixel spacing = 0.45 mm × 0.45 mm, slice number = 18, slice thickness = 6 mm, matrix size = 512 × 512 × 18), T2‐weighted FLAIR (TE = 120 ms, TR = 7000 ms, flip angle = 90°, FOV = 230 mm × 230 mm, pixel spacing = 0.6 mm × 0.6 mm, slice number = 18, slice thickness = 6 mm, matrix size = 384 × 384 × 18) and time‐of‐flight (TOF) MRA (TE = 3.5 ms, TR = 23 ms, flip angle = 18°, FOV = 210 mm × 210 mm, pixel spacing = 0.375 mm × 0.375 mm, slice number = 112, slice thickness = 0.8 mm, matrix size = 560 × 560 × 112).

WMH lesions were segmented automatically by the lesion prediction algorithm as implemented in the Lesion Segmentation Toolbox (www.statistical‐modelling.de/lst.html) for SPM (https://www.fil.ion.ucl.ac.uk/spm/). WMH were segmented on axial T2‐FLAIR images, which are considered the most sensitive sequence for identifying WMH because they suppress cerebrospinal fluid signal and highlight periventricular and deep white matter lesions with high contrast. We therefore relied on T2‐FLAIR alone to ensure consistent and optimal lesion detection across all participants, and avoid potential inconsistencies from multimodal segmentation. For each individual, T2‐FLAIR images were registered to the T1‐weighted images using an affine transformation and the T1‐weighted images were registered to the MNI brain atlas by non‐linear transformation. Then, two transformations were concatenated to transform the T2‐FLAIR images and corresponding WMH lesions images into the reference space [11]. WMH lesions were divided into PWMH or DWMH according to the distance from the lateral ventricles (> 10 mm was considered to be deep WMH) [12].

### 2.3 Arterial Flow Simulation

The cerebral arterial blood flow was simulated using a patient‐specific simplified hemodynamic model which has been applied in both healthy subjects and those with gliomas [8, 9]. In brief, the steps were as follows (Figure S1): (1) Cerebral vessel segmentation was completed on MRA images using the previously described methods [13]. The vessels were then reconstructed into 3D space to allow interactive manual selection of inflow vessel branches, including internal carotid and basilar artery. All segmentation results were manually checked to ensure that at least all secondary branches were completely segmented (M2/P2/A2). (2) The centerlines of vessels were extracted using the Skeleton 3D toolbox [20]. The topological connections among vascular branches were organized by evaluating the adjacency of centerline points. The inflow branches were manually identified, while all outflow branches were automatically obtained through the topological connections. (3) The flow and pressure drop of each vascular branch were related according to the Hagen–Poiseuille equation. By solving these equations, the flow and pressure drop of each vascular branch were obtained. To accomplish this, both the inflow and outflow pressure of each branch were served as unsolved variables and three sets of boundary conditions were established. For inlet, the mean arterial pressure was served as a boundary condition for a virtual vascular branch which was generated based on all the inflow branches (including the internal carotid artery and the basilar artery) and distributed the blood flow according to cross‐sectional area [8, 9]. For outlet, a patient‐specific structured tree was built for each outflow branch until the diameter of the terminal branches in the structured tree was less than 0.1 mm [21]. For bifurcation, the incoming flow was equal to the outgoing flow and pressure was assumed to be continuous. Since all the governing equations were linear, the flow features for each vascular branch were directly obtained using matrix inversion methods. Based on the pressures at the inflow and outflow of each branch, the mean pressure and pressure drop were calculated. More details were in the Supporting Information and all data processing was performed on MATLAB (version 2022a; MathWorks, Natick, Massachusetts, USA).

### 2.4 Arterial Feature Analysis

According to the simulation process, three flow features of each vascular branch, including flow rate, mean pressure (the mean of inlet and outlet pressures), and pressure drop (the difference between inlet and outlet pressures), were quantified. The distances between each WMH lesion and all terminal arterial branches visible in TOF images were obtained after linear registration of T2 FAIR images to TOF images. Here, terminal arterial branches were defined as all terminal outlet branches with diameters smaller than the mean diameter of all vascular branches to exclude potentially larger outlet vascular branches. The distance between WMH lesions and arterial branches was defined as the shortest Euclidean distance from the lesion boundary to the vascular branch boundary. Moreover, a threshold was established to define adjacent‐to‐lesion terminal arterial branches (ALTABs), which was defined as a terminal arterial branch visible in TOF images within a certain distance around the lesions. The threshold distance was determined as the mean shortest Euclidean distance from all WMH lesions to their respective nearest arterial branch.

### 2.5 Evaluation Model Performance

To validate the performance of the simplified hemodynamic model, this study employed Flow‐MRI to measure blood flow in critical intracranial vascular branches and compared it with simulation results. Data were derived from 18 healthy volunteers (mean age, 30.1 ± 5.5 years, 72.2% female), with both TOF MRA and Flow‐MRI images being concurrently collected. The protocol of Flow‐MRI was as follows: TE = 3.6 ms, TR = 8.0 ms, flip angle = 10°, matrix size = 224 × 224 × 20, voxel size = 0.94 mm × 0.94 mm × 2 mm, VENC = 120 cm/s. Flow rates in the left and right internal carotid arteries, middle cerebral arteries, posterior cerebral arteries, and basilar artery, considered as measurement standards, were obtained through the GTFlow tools (www.gyrotools.com). Three different planes were manually selected on each arterial segment, and the mean value was calculated to represent the flow rate of this artery. Subsequent results from simulations using TOF‐MRA were compared with those from Flow‐MRI.

### 2.6 Statistical Analysis

Multivariable linear regression analysis was used to assess the association between WMH and cerebral arterial flow features. The flow features of all arterial branches for each individual were averaged initially, and the relationships between these features and WMH were analyzed. Subsequently, following the calculation of ALTABs based on the distance threshold, a comparison was undertaken between the flow features of ALTABs and other terminal branches. The associations between the mean flow features of all ALTABs and the total WMH volume were also analyzed. Furthermore, correlation analysis at the lesion level was conducted based on all lesions across all individuals. Multivariable models were adjusted for potential confounding variables including age, sex, and cerebrovascular risk factors, including cigarette use, alcohol consumption, diabetes, hyperlipidemia, hypertension, and history of cardiovascular disease (coronary artery disease, atrial fibrillation, heart failure, or heart valve disease).

Due to the skewed distribution of WMH lesion volume, logarithmic transformation was applied. The continuous variables were presented as mean and SD or as median and interquartile range. For categorical variables, frequencies and proportions were presented. A two‐sample *t*‐test was applied to evaluate group differences. All tests were two‐sided, and a *p* value of < 0.05 was considered significant. Moreover, random selection of a tenth of the data from all patients as subgroups, and repetition of the statistical analysis were completed to assess the reproducibility of the statistical results. All statistical analyses were undertaken using MATLAB (version 2022a; MathWorks, Natick, Massachusetts, USA).

## 3 Results

### 3.1 Individual Characteristics

**FIGURE 1:**
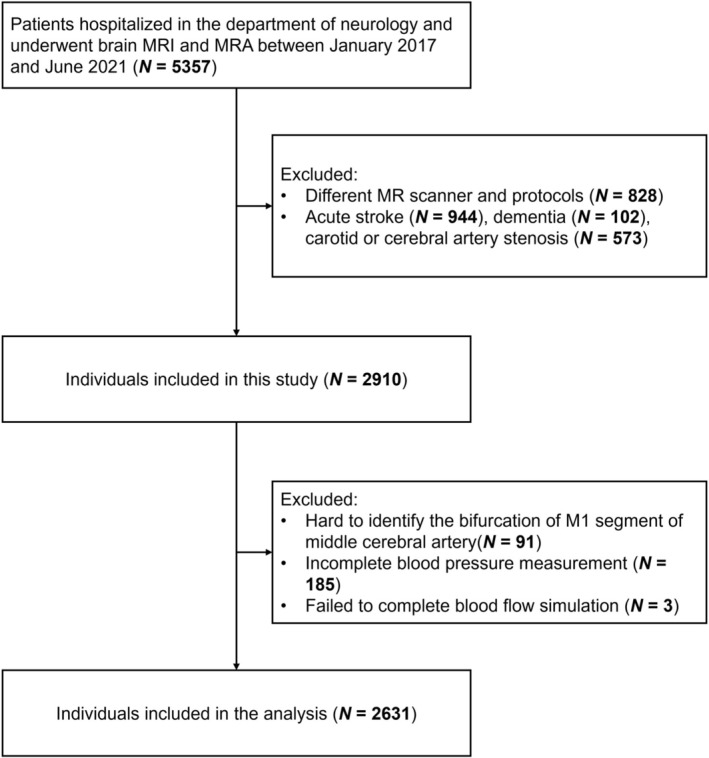
Flow chart of enrollment in the current study.

The individual characteristics are presented in Table 1. Figure 1 summarizes the enrollment process of the current study. A total of 2910 individuals were initially included; of these, 94 individuals with poor vascular segmentation quality and 185 individuals with incomplete BP measurement were excluded. Therefore, 2631 individuals were included in the subsequent analysis. The mean (SD) age was 68.5 (11.1) years, and 1324 (50.3%) individuals were female.

**TABLE 1:** Individual characteristics.

Participants, No.	2631
Demographic	
Age, mean (SD), years	68.5 (11.1)
Female, No. (%)	1324 (50.3)
Smoking, No. (%)	360 (13.7)
Alcohol consumption, No. (%)	181 (6.88)
Diabetes diagnosis, No. (%)	761 (28.9)
Hyperlipidemia, No. (%)	476 (18.1)
Hypertension, No. (%)	1731 (65.8)
History of cardiovascular disease, No. (%)	456 (17.3)
White matter hyperintensities (WMH)	
Volume, median (IQR), mL	6.58 (18.9)
Periventricular WMH volume, median (IQR), mL	6.12 (18.5)
Deep WMH volume, median (IQR), mL	0.24 (0.46)
Hemodynamic features^a^	
Mean flow rate, mean (SD), mL/min	7.86 (1.40)
Mean pressure, mean (SD), mmHg	82.4 (7.15)
Mean pressure drop, mean (SD), mmHg	0.51 (0.09)

^a^ The hemodynamic features refer to the mean values of the hemodynamic features of all arterial branches visible in MRA images.

### 3.2 Association Between Cerebral Arterial Flow Features and WMH

In general, higher mean flow rate and pressure were found to be associated with increased total WMH volume after adjusting for age, sex and risk factors (mean flow rate: *β* = 0.10, 95% CI: 0.06–0.14, *p* < 0.001; mean pressure: *β* = 0.03, 95% CI: 0.02–0.04, *p* < 0.001) as shown in Table 2. When WMHs are further divided into PWMHs and DWMHs, a significant association was identified between mean flow rate and PWMH volume (*β* = 0.09, 95% CI: 0.05–0.13, *p* < 0.001), and DWMH volume was found to be associated with mean pressure drop, instead of the mean flow rate (*β* = 1.04, 95% CI: 0.11–1.98, *p* = 0.03). These results were adjusted for age, sex and risk factors.

**TABLE 2:** Association between cerebral arterial flow pattern and white matter hyperintensity.

	Bivariable model		Adjusted model 1		Adjusted model 2	
	*β* (95% CI)	*p*	*β* (95% CI)	*p*	*β* (95% CI)	*p*
Total WMHV						
Mean flow rate, mL/min	0.13 (0.08–0.19)	< 0.001	0.13 (0.09–0.17)	< 0.001	0.10 (0.06–0.14)	< 0.001
Mean pressure, mmHg	0.05 (0.04–0.06)	< 0.001	0.04 (0.03–0.04)	< 0.001	0.03 (0.02–0.04)	< 0.001
Mean pressure drop, mmHg	1.80 (0.98–2.63)	< 0.001	1.03 (0.39–1.67)	0.002	0.40 (−0.24–1.04)	0.22
PWMHV						
Mean flow rate, mL/min	0.13 (0.07–0.18)	< 0.001	0.12 (0.08–0.17)	< 0.001	0.09 (0.05–0.13)	< 0.001
Mean pressure, mmHg	0.05 (0.04–0.06)	< 0.001	0.04 (0.03–0.05)	< 0.001	0.03 (0.02–0.04)	< 0.001
Mean pressure drop, mmHg	2.00 (1.09–2.9)	< 0.001	1.15 (0.43–1.88)	0.002	0.50 (−0.23–1.22)	0.18
DWMHV						
Mean flow rate, mL/min	0.08 (0.01–0.14)	0.02	0.07 (0.02–0.13)	0.01	0.04 (−0.02–0.10)	0.19
Mean pressure, mmHg	0.04 (0.02–0.05)	< 0.001	0.03 (0.02–0.04)	< 0.001	0.02 (0.01–0.03)	< 0.001
Mean pressure drop, mmHg	2.24 (1.2–3.28)	< 0.001	1.68 (0.76–2.61)	< 0.001	1.04 (0.11–1.98)	0.03

*Note:* Model 1 adjusted for age and sex; model 2 adjusted for age, sex and cerebrovascular risk factors including cigarette use, alcohol consumption, diabetes, hyperlipidemia, hypertension, and history of cardiovascular disease.

Abbreviations: DWMHV, deep white matter hyperintensity lesion volume; PWMHV, periventricular white matter hyperintensity lesion volume; WMHV, white matter hyperintensity lesion volume.

These associations were further examined in hypertensive and non‐hypertensive individuals (Tables S1 and S2). In the multivariable adjusted model, these associations persisted in both hypertensive and non‐hypertensive individuals. However, in the bivariable model, all three flow features were correlated with the total volume of WMH in non‐hypertensive individuals (mean flow rate: *β* = 0.21, 95% CI: 0.10–0.32, *p* < 0.001; mean pressure: *β* = 0.07, 95% CI: 0.05–0.09, *p* < 0.001; mean pressure drop: *β* = 4.17, 95% CI: 2.35–5.99, *p* < 0.001), whereas only mean pressure was correlated with the total WMH volume in hypertensive individuals (*β* = 0.02, 95% CI: 0.008–0.03, *p* = 0.001).

### 3.3 ALTAB Flow Features and WMH

**FIGURE 2:**
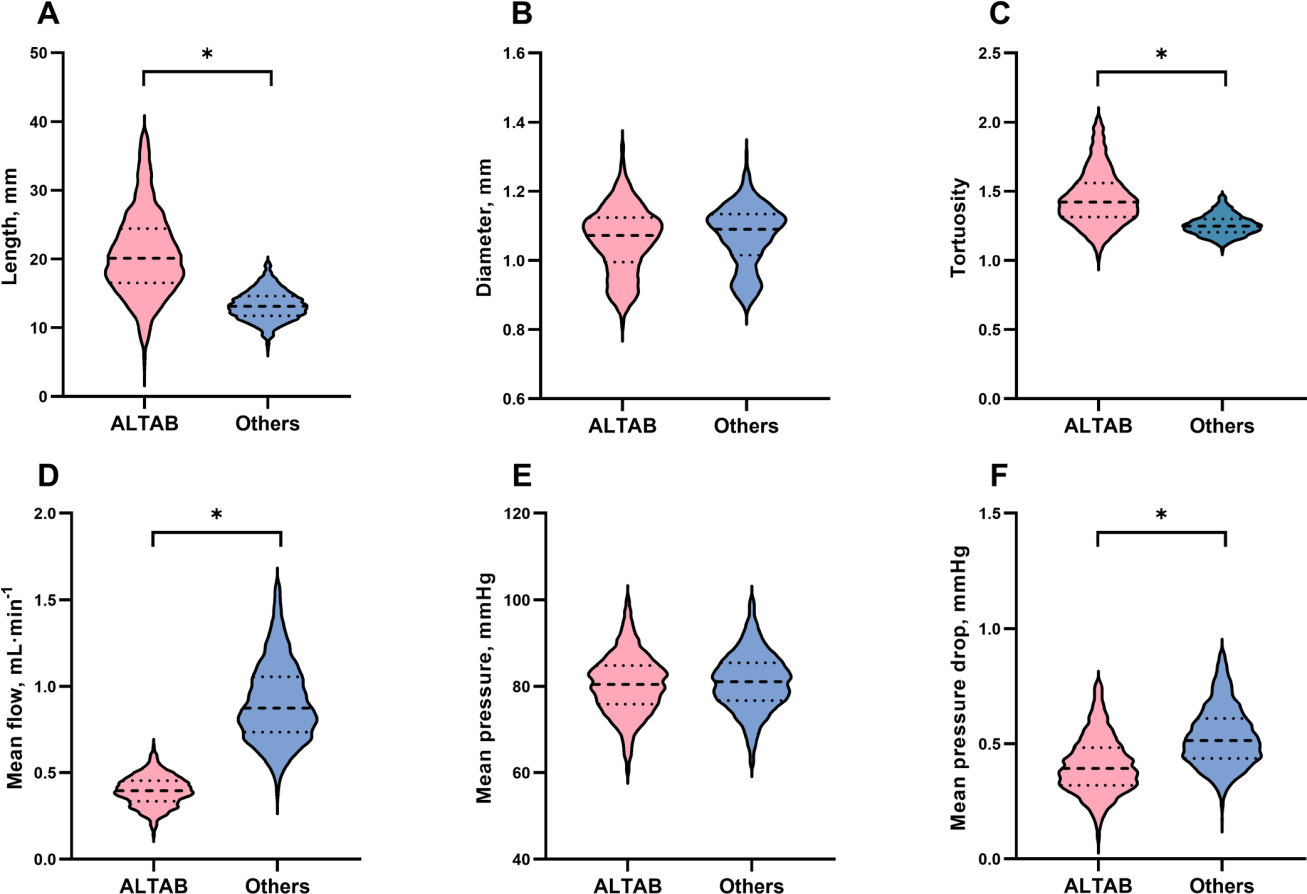
(A–F) Difference between adjacent lesion terminal arterial branches and other arterial branches. The red section represents adjacent‐to‐lesion terminal arterial branches (ALTAB, meaning the terminal arterial branches located adjacent to a WMH lesion), while the blue section represents other terminal branches. The asterisk indicates a significant difference with *p* < 0.0083 (Bonferroni correction: 0.05/6≈0.0083).

Based on the histogram of distances between all WMH lesions and corresponding nearest terminal vessel branch shown in Figure S2, a mean value of 13 mm from the lesion boundary was used as the demarcation for ALTAB. As presented in Figure 2, compared to other terminal arterial branches, ALTABs exhibited greater length (21.7 ± 8.21 mm vs. 13.3 ± 2.35 mm, *p* < 0.001) and tortuosity (1.52 ± 0.39 mm vs. 1.26 ± 0.11 mm, *p* < 0.001). In terms of flow features, ALTABs had lower mean flow rates (0.40 ± 0.09 mm vs. 0.90 ± 0.25 mm, *p* < 0.001) and pressure drops (0.42 ± 0.16 mm vs. 0.54 ± 0.15 mm, *p* < 0.001). The comparison of features between ALTABs and other terminal branches under different thresholds that are used to categorize terminal vascular branches, is shown in Figures S3 and S4. As the threshold varied from 6 to 20 mm, the feature differences between ALTABs and other terminal branches remained relatively consistent, suggesting that the potential inherent differences that existed in ALTABs might be minimally affected by the selection of the threshold.

Association between ALTAB flow features and WMH volume was presented in Table 3. There were significant associations between total WMH volume and the mean flow features of all corresponding ALTABs. Specifically, higher mean ALTAB flow rate was correlated with greater WMH volume (*β* = 3.43, 95% CI: 2.69–4.17, *p* < 0.001), higher mean ALTAB pressure was correlated with greater WMH volume (*β* = 0.06, 95% CI: 0.05–0.07, *p* < 0.001), and lower mean ALTAB pressure drop was correlated with greater WMH volume (*β* = −1.35, 95% CI: −1.77 to −0.93, *p* < 0.001). These associations remained significant after adjusting for age, sex and other cerebrovascular risk factors.

**TABLE 3:** Association between flow features of adjacent‐to‐lesion terminal arterial branches and white matter hyperintensity.

	Bivariable model		Adjusted model 1		Adjusted model 2	
	*β* (95% CI)	*p*	*β* (95% CI)	*p*	*β* (95% CI)	*p*
ALTAB mean flow, mL/min	3.43 (2.69–4.17)	< 0.001	2.93 (2.35–3.52)	< 0.001	2.67 (2.1–3.25)	< 0.001
ALTAB mean pressure, mmHg	0.06 (0.05–0.07)	< 0.001	0.05 (0.04–0.05)	< 0.001	0.04 (0.04–0.05)	< 0.001
ALTAB mean pressure drop, mmHg	−1.35 (−1.77 to 0.93)	< 0.001	−0.82 (−1.16 to −0.49)	< 0.001	−0.86 (−1.19 to −0.53)	< 0.001

*Note:* Model 1 adjusted for age and sex; model 2 adjusted for age, sex and cerebrovascular risk factors including cigarette use, alcohol consumption, diabetes, hyperlipidemia, hypertension, and history of cardiovascular disease.

Abbreviation: ALTAB, adjacent‐to‐lesion terminal arterial.

### 3.4 Lesion‐Based Analysis

**FIGURE 3:**
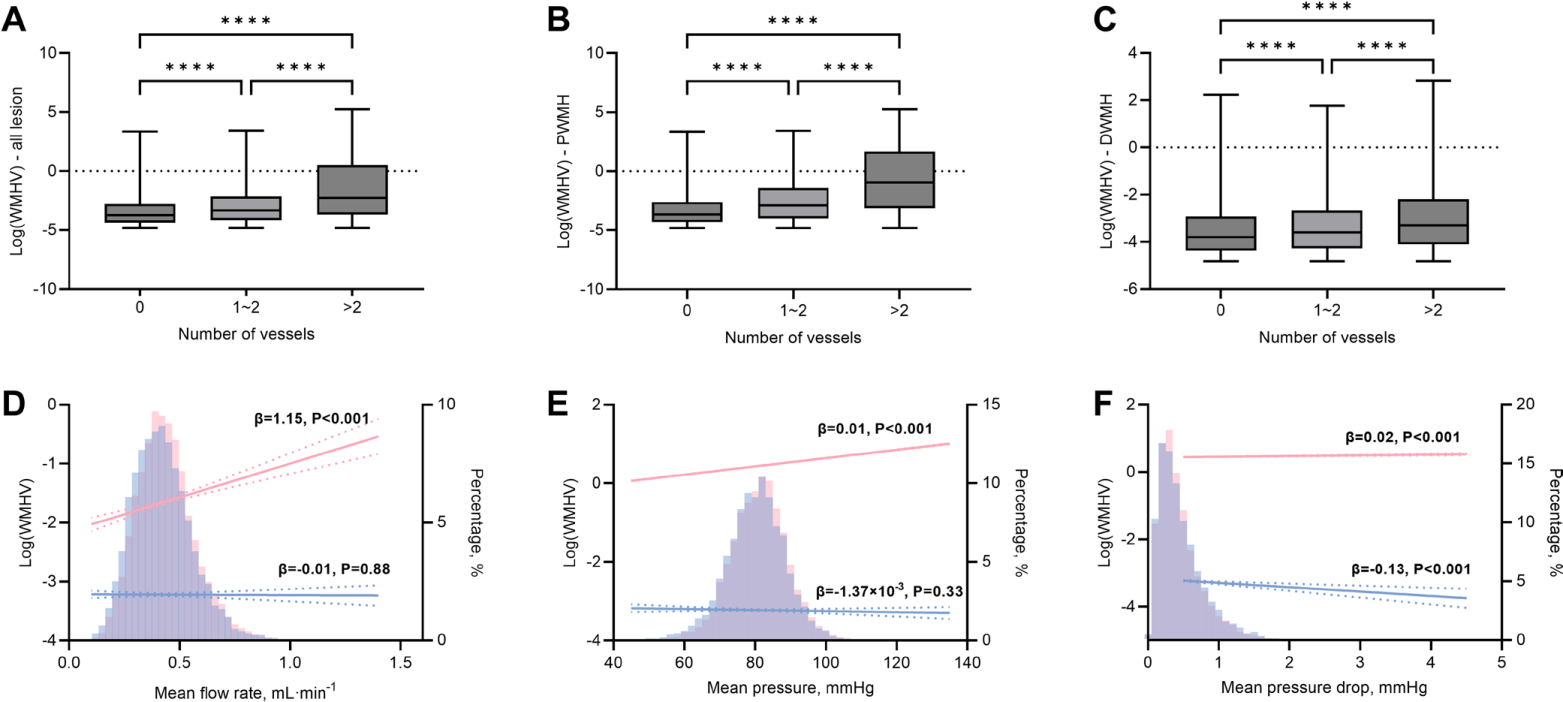
Lesion‐based analysis of association between adjacent lesion terminal arterial branches and white matter hyperintensity. Comparation of adjacent‐to‐lesion terminal arterial branches (ALTAB, meaning the terminal arterial branches located adjacent to a WMH lesion) number with: (A) total white matter hyperintensity (WMH) volume, (B) periventricular WMH volume, and (C) deep WMH volume. (D–F) The association of ALTAB flow features with WMH volume. The relationships (indicated by a bold line) and 95% CIs (dotted line) are derived from linear regression models. Histograms of flow features are displayed as solid bars. The red section represents ALTAB of periventricular WMH, while the blue section represents ALTAB of deep WMH. ****P < 0.0001.

Further investigation was conducted on a lesion‐by‐lesion basis to study the relationship between ALTBAs flow features and WMH as shown in Figure 3. Compared to lesions without ALTABs (where the distance between the lesion and all TOF MRA visible terminal vascular branches was greater than 13 mm), lesions with ALTABs had larger volumes, and the volume of the lesion increases with a greater number of ALTABs (log‐converted WMH volume of no ALTABs vs. one ALTAB vs. two and more ALTABs: −3.43 ± 1.18 vs. −3.10 ± 1.38 vs. −1.96 ± 2.31). Their relationships also remained in both PWMH and DWMH.

Figure 3 shows the lesion‐based association between ALTAB flow features and WMH volume. For PWMH, similar to the aforementioned associations, high ALTAB flow rate, mean pressure and pressure drop were correlated with high PWMH volume (flow rate: *β* = 1.15, 95% CI: 0.84–1.46, *p* < 0.001; mean pressure: *β* = 0.0105, 95% CI: 0.0103–0.0107, *p* < 0.001; pressure drop: *β* = 0.02, 95% CI: 0.01–0.03, *p* < 0.001). In contrast, for DWMH, only low ALTAB pressure drop was correlated with high DWMH volume (*β* = −0.13, 95% CI: −0.20 to −0.06, *p* < 0.001).

### 3.5 Model Performance

Cerebral arterial flow rate between Flow‐MRI and simulation is shown in Figure S5. A positive correlation of arterial flow rate between Flow‐MRI and simulation was observed (*R* ^2^ = 0.497, *p* < 0.001), suggesting that the simulation results from a simplified model, to a certain extent, could characterize the real blood flow. Tables S3–S5 show the outcomes of 10 repeated random selections and corresponding correlation results, demonstrating that the significant correlation between arterial flow features and WMH remains significant even in smaller subsets. These findings indicate that the results in the current study have a good degree of reproducibility.

## 4 Discussion

In this study, cerebral arterial flow features including flow rate, mean pressure and pressure drop were quantified based on the patient‐specific simplified model. Significant associations of all three arterial flow features with WMH volume were identified suggesting abnormal cerebral hemodynamics might increase the liability of WMH to develop. Moreover, associations of PWMH and DWMH with flow features were not consistent. Such findings are not surprising given the distinct anatomical and vascular characteristics of the general deep white matter or periventricular white matter that require differential perfusion profiles and vascular morphologies for their specialized functions. The current study provides preliminary data on the impact of cerebral arterial blood flow on WMH, implying the morphology and corresponding flow features of proximal cerebral arteries might be one of the targets for addressing WMH.

Both arterial flow rate and pressure were associated with WMH in the current study. Regarding the impact of blood flow variation on WMH, several studies had noted a negative correlation between cerebral perfusion blood flow and WMH volume [22, 23]. A longitudinal study indicated that a lower baseline cerebral perfusion blood flow was associated with a greater volume of newly developed WMH lesions [24]. However, the impact of surrounding arterial flow characteristics on WMH was unclear. Results from ultrasonic studies of intracranial arterial flow features were rather inconclusive. A negative correlation between the flow velocity of the intracranial segment of the carotid artery and WMH lesion volume has occasionally been reported [25], while the other study noted a positive relationship between increased fluctuations in the blood flow of the intracranial segment of the carotid artery and WMH lesion volume [26]. The current study found a positive correlation between WMH burden and the mean flow rate of all TOF‐visible cerebral arterial branches. This finding might not contradict the well‐established inverse relationship between cerebral perfusion and WMH severity. Cerebral perfusion measures reflect the blood flow achievable in the microvascular networks of tissue. We assumed that chronic microvascular hypoperfusion in WMH regions increases distal resistance and stimulates upstream dilation of larger arteries, resulting in higher flow in major TOF‐visible vessels as a compensatory response. Consistent with this, high‐resolution MRI has shown intracranial arterial dilation in patients with extensive WMH despite no proximal stenosis, suggesting an adaptive large‐artery response to distal ischemia [27]. However, if the downstream small‐vessel network is diseased or rarefied, this compensatory increase in large‐artery flow does not translate into adequate tissue perfusion, leaving the affected white matter chronically under‐supplied, a conclusion further supported by our finding that ALTABs carry significantly lower flow than comparable distal terminal arteries elsewhere in the brain. Similar paradoxical findings have been reported in other cohorts [28, 29]. Existing research shows that elevated large‐artery flow velocities have been associated with greater WMH load, higher stroke risk, and more hemorrhagic complications, reflecting vascular stress and impaired autoregulation rather than benign hyperemia [28, 29]. Taken together, these data support the view that the elevated arterial flow observed might represent an incomplete compensation for microvascular dysfunction rather than true hyperperfusion of the white matter. Additionally, we acknowledge that alternative mechanisms beyond compensatory hemodynamic responses might account for the observed pattern. As a cross‐sectional study, it is possible that our enrollment influenced the findings. Individuals with both low global arterial flow and low lesion‐adjacent flow might be more likely to suffer symptomatic stroke, severe cerebrovascular events, or increased mortality, and therefore would not be represented in our study cohort. As a result, the remaining sample might include more individuals who exhibit the hemodynamic pattern of higher global inflow alongside low distal lesion‐adjacent perfusion, thereby producing the observed results. Further prospective studies are warranted to confirm this compensatory mechanism and its clinical implications.

Regarding the influence of blood pressure on WMH, the association between cuff blood pressure and WMH has been studied in both cross‐sectional and longitudinal studies, revealing a relationship between increased blood pressure and a greater WMH lesion volume [30, 31]. Similarly, a positive correlation between increased mean pressure of all TOF‐visible cerebral arterial branches and WMH volume was observed in this study. These results add to the evidence that blood pressure control might benefit patients with small vessel disease. However, the association of flow features and WMH was not fully consistent between hypertensive and non‐hypertensive individuals, with more changes in the flow characteristics associated with WMH in the non‐hypertensive individuals. This might be due to accumulated variations in cerebral arterial flow, cerebral perfusion, as well as vascular wall among hypertensive individuals [32, 33], resulting in arterial flow features no longer being one of the major factors affecting WMH. Further studies from different disciplines are required to clarify such findings.

This study used 13 mm as the threshold to define ALTAB. Although it was chosen empirically from our data, anatomical data suggest this distance is physiologically reasonable. The cerebral white matter is supplied by long medullary arteries that descend from pial vessels and act as end‐arteries with few anastomoses [34]. Short cortical arterioles penetrate about 5–10 mm, whereas deep medullary branches can extend 20–50 mm to the periventricular region, creating distal “watershed” zones about 3–13 mm from the ventricles that are especially vulnerable to hypoperfusion [35, 36]. Moreover, infarcts from occlusion of a single medullary artery are typically 10 mm in diameter [37]. These observations indicate that a vessel within 13 mm of a WMH lies well inside the range of its potential perfusion field.

This study identified significant differences in the morphology and flow features between terminal arterial branches near WMH (ALTAB) and distant terminal arterial branches. Most of the previous studies focused on the overall characteristics of small vessels. For instance, a general alteration in the hemodynamics of brain arterial branches during the progression of glioma was observed [8]. Disturbed middle cerebral artery flow velocity has been linked to the occurrence of stroke [28, 29]. Such studies, however, have not paid attention to the distinctions between adjacent and distant vessels to the lesions and were not able to uncover regional variations in the interaction between the lesion and the vasculature, thus preventing the identification of vascular features specific to each local lesion. The current study found that arterial branches near WMH lesions (ALTAB) exhibit lower blood flow rates and pressure, suggesting the potential for insufficient blood supply in the areas served by these branches. These findings might facilitate early identification of white matter lesions. Moreover, ALTAB was noted to have greater tortuosity, consistent with the finding that vessels around the tumor were more tortuous and again implying a possible interaction between the lesion and surrounding vessels [38]. Notably, despite ALTABs in this study being only spatially closer to WMH lesions and might not represent the actual arteries supplying the WMH lesions, their spatial proximity might facilitate the transfer of arterial pulsatile damage to the lesion area, potentially influencing the development of the lesion.

In lesion‐based analysis, the current study observed a greater number of ALTABs surrounding larger WMH lesions. This could be a result of the close distance of the lesions to the arterial vessels, dictated by larger volume. Besides, it might also be associated with factors including collateral circulation to compensate for long‐term ischemia. The establishment of collateral circulation might affect the flow velocity of the surrounding arteries [39], thereby influencing their visualization in TOF MRA which depends on flow‐related enhancement [40]. Furthermore, an inconsistent impact of ALTAB flow features on the volume of PWMH and DWMH lesions was observed. ALTAB flow features were more closely associated with the volume of PWMHs than with DWMHs. This result added to the existing evidence that spatially distinct clusters of WMHs might be involved in differing pathological processes [41, 42]. A previous study has shown that reduced cerebral blood flow was associated with the increased volume of PWMH but not DWMH [43]. Moreover, due to the structural differences in the supplying arteries, PWMH and DWMH exhibit varying degrees of sensitivity to fluctuations in blood pressure [44]. Future research should pay more attention to the diverse locations of WMHs.

Our findings are consistent with previous research of CSVD pathophysiology, which emphasizes arterial stiffening, impaired autoregulation, endothelial injury, and chronic hypoperfusion. Increased large‐artery stiffness raises pulsatile pressure and flow load on downstream arterioles, promoting blood–brain barrier disruption, perivascular inflammation, and progressive white matter injury [45, 46]. As distal resistance rises, proximal arteries may dilate and increase flow to maintain perfusion, producing the paradox we observed of higher large‐artery flow concurrent with reduced microvascular perfusion [47]. Such compensatory but ultimately inadequate responses have been described in imaging and physiological studies linking pulse‐wave velocity, transcranial Doppler pulsatility, and MRI markers of SVD [45, 48]. Nevertheless, large‐scale investigations that quantify cerebral hemodynamics in CSVD cohorts remain limited, highlighting the value of approaches like ours that could assess flow and pressure features across broad clinical populations.

The simulation model in this study was a simplified form of the one‐dimensional hemodynamics model. This model averaged the temporal variation in flow velocity and pressure, and utilized the steady‐flow control equations to represent the relationship between blood flow velocity and pressure, thereby avoiding the solution of Navier–Stokes equations [8]. Moreover, this model only requires vascular imaging and standard cuff blood pressure as boundary conditions, which were relatively easy to obtain in most clinical centers, making it promising for rapid assessment of cerebrovascular dynamics on large‐scale clinical datasets. A previous study has compared the simulation results between this zero‐dimensional model and the three‐dimensional model, and a strong linear correlation was observed [9]. This model has also been employed to compare the blood flow variation in patients with brain gliomas at different stages [8]. Other flow or pressure‐based methods have also been applied to cerebrovascular research. Transcranial Doppler ultrasonography measures flow velocity in major intracranial arteries and provides indices such as the pulsatility index, which correlates with small‐vessel disease burden [48], but it is operator‐dependent and limited to large basal vessels. High‐fidelity CFD can reconstruct three‐dimensional vascular geometries to calculate wall shear stress and pressure gradients with high spatial resolution [49], yet it is computationally intensive and rarely feasible in large patient cohorts. Recent patient‐specific CFD analyses of the ophthalmic artery demonstrated correlation between arterial diameter and cerebral small vessel disease but involved only a few cases [50]. These approaches illustrate the trade‐off between physiologic detail and scalability. The present simplified model balances physiological relevance with clinical practicality, enabling network‐level estimates of both flow and pressure from routinely available imaging and blood pressure data. Despite these advances, large‐scale studies assessing cerebral hemodynamics specifically in patients with cerebral small vessel disease remain very limited, underscoring the need for methods such as ours that can be applied across broad clinical populations.

There are several limitations to be addressed. First, this study utilized only a simplified hemodynamic model to simulate cerebral arterial blood flow features, which could not encompass the temporal trends of blood flow. While employing more sophisticated hemodynamic models could provide a more accurate representation of the arterial flow features, it necessitates precise boundary conditions, deeper specialized knowledge, as well as substantial resources. This might not be feasible for large‐scale clinical image data. More efficient and rapid hemodynamic assessment methods are needed to address this issue.

Second, single mean arterial pressure was used as the input boundary condition, which might not capture patient‐specific autoregulatory capacity, differences in blood pressure fluctuations, or variations in arterial wall compliance. More sophisticated models incorporating patient‐specific blood pressure variability or vessel elasticity as the boundary condition could improve simulation accuracy.

Third, the configuration of the (CoW) was not included in the analysis. Variations of CoW could significantly affect cerebral blood flow distribution and collateral pathways, which in turn could influence WMH development. However, determining the functional status of the CoW from time‐of‐flight MRA alone was challenging, as flow direction and small communicating arteries were difficult to determine in our images. Future studies should incorporate advanced imaging, such as dedicated flow MRI or high‐resolution MRA of the CoW to capture the anatomical differences, as they could have a significant influence on collateral circulation.

Fourth, the causal relationships between flow features and WMH cannot be established in this study because of its retrospective and observational nature. The causal interaction between arterial flow and small vessel disease remains uncertain and requires prospective, longitudinal research to clarify.

Fifth, in addition to the risk factors included in this study, multiple risk factors such as exercise, diet, sleep, and mental health are potential confounders of our results. Unfortunately, those factors are not considered in the current study given its retrospective nature. A comprehensive evaluation of the associations of risk factors and WMH is needed.

Sixth, an important limitation concerned selection bias due to our recruitment of hospital‐based patients excluding individuals with cerebrovascular disease such as stroke. This might have several consequences: (1) the sample might overrepresent individuals with CSVD which could exaggerate associations between arterial flow features and WMH in a more general population; (2) by excluding individuals with stroke or other major vascular events, we might under‐sample individuals with severe hemodynamic disturbance, reducing the range of both WMH burden and arterial flow abnormalities, which could attenuate some relationships; and (3) hospital patients often differ from community‐based individuals in comorbidity burden, treatment exposure, and diagnostic screening, which might influence both arterial flow and WMH measurements. Hence, the generalizability of our findings to broader, healthy or mixed populations is limited and effect sizes might not be directly applicable outside clinical settings.

Seventh, the binary distinction of PWMH and DWMH in this study was as an initial, simplified spatial categorization and therefore might be dated and have limitations. Treating the distance from the ventricles as a continuous variable might provide a more detailed understanding of how WMH location influences or relates to vascular features. Moreover, alternative descriptors, such as voxel‐wise mapping of WMH frequency or tract‐based spatial assessments, might capture territorial patterns of WMH more richly than simple binary distinction.

Finally, although a large sample was used, most of the individuals were elder Chinese residing in Shanghai. Moreover, correlation analyses in this study were largely exploratory without multiple comparisons correction. As a result, the findings presented in the current study might not be pronounced in the global population and should also be interpreted with caution. Future studies involving multi‐racial individuals are required to establish the association between cerebral arterial flow features and small vessel disease.

## 5 Conclusions

In conclusion, this study identified a two‐level pattern between cerebrovascular hemodynamics and WMH load. Globally, higher flow in major TOF‐visible arteries suggests systemic compensation, while locally, terminal branches adjacent to WMH carry lower flow, indicating focal hypoperfusion. These findings imply a mismatch between increased upstream inflow and inadequate downstream delivery, highlighting impaired autoregulation in CSVD. Clinically, noninvasive assessment of large‐vessel hemodynamics with routine TOF MRA and a simplified model might help identify individuals at risk for WMH‐related cognitive decline or stroke and underscore the need to evaluate both macrovascular and microvascular factors in patient care. Caution is warranted because this retrospective study cannot establish any causal relationship between cerebral arterial flow features and small vessel disease. Future longitudinal and mechanistic studies will be required to determine the temporal sequence and clarify whether altered arterial flow is a cause or a consequence of small‐vessel pathology.

## Funding

This work was supported by the National Natural Science Foundation of China (No. 82402233, 82271956 and 62331021), National Key R&D Program of China (No. 2023YFF1204804), and Shanghai Municipal Health Commission Clinical Research Special Program (No. 202240126).

## Ethics Statement

This retrospective, observational study was approved by the Ethics Committee of Yueyang Hospital of Integrated Traditional Chinese and Western Medicine, Shanghai University of Traditional Chinese Medicine in accordance with the Declaration of Helsinki (No. 2020‐060). The study used only previously collected data, with no additional tests or interventions undertaken on any participants and thus would inflict no impact on patient care or outcomes. Therefore, the written informed patient consent was not included in this study.

## Conflicts of Interest

The authors declare no conflicts of interest.

## Supporting information


**Data S1:** cns70673‐sup‐0001‐DataS1.docx.

## Data Availability

The data that support the findings of this study are available from the corresponding author upon reasonable request.
