# Supplementary material for: Distinct Hemodynamic and Morphological Characteristics of Arteries Adjacent to White Matter Hyperintensities
Source: CNS Neurosci Ther. 2025 Nov 24;31(11):e70673. doi: 10.1111/cns.70673 (PMC12641447; doi:10.1111/cns.70673)
Supplement: Supplementary file 1 — Data S1: cns70673‐sup‐0001‐DataS1.docx. [file CNS-31-e70673-s001.docx]

**Supplementary Material Content**

**This supplementary material includes:**

Supplementary Method

Supplementary Figure S1. Schematic representation of the simplified hemodynamic model implementation.

Supplementary Figure S2. Histogram of distance between white matter hyperintensity and the nearest terminal arterial branch.

Supplementary Figure S3. Morphology between Adjacent-to-Lesion Terminal Arterial Branches and other branches under different threshold.

Supplementary Figure S4. Flow features between Adjacent-to-Lesion Terminal Arterial Branches and other branches under different threshold.

Supplementary Figure S5. Cerebral arterial flow rate between Flow-MRI and simulation.

Supplementary Table S1. Association between cerebral arterial flow pattern and white matter hyperintensity in hypertensive individuals.

Supplementary Table S2. Association between cerebral arterial flow pattern and white matter hyperintensity in non-hypertensive individuals.

Supplementary Table S3. Association between cerebral arterial flow pattern and white matter hyperintensity in subgroups.

Supplementary Table S4. Association between flow features of Adjacent-to-Lesion Terminal Arterial Branches and white matter hyperintensity in subgroups.

Supplementary Table S5. Two sample t-test between Adjacent-to-Lesion Terminal Arterial Branches and other arterial branches in subgroups.

**Supplementary Methods**

***Brain MRI and Analysis***

The MRI protocol included T1-weigthed structural imaging (TE=2.3ms, TR=250ms, flip angle=75°, FOV=230mm×230mm, pixel spacing=0.45mm×0.45mm, slice number=18, slice thickness=6mm, matrix size=512×512×18), T2-weighted FLAIR (TE=120ms, TR=7000ms, flip angle=90°, FOV=230mm×230mm, pixel spacing=0.6mm×0.6mm, slice number=18, slice thickness=6mm, matrix size=384×384×18) and time-of-flight (TOF) MRA (TE=3.5ms, TR=23ms, flip angle=18°, FOV=210mm×210mm, pixel spacing=0.375mm×0.375mm, slice number=112, slice thickness=0.8mm, matrix size=560×560×112).

WMH lesions were segmented automatically by the lesion prediction algorithm as implemented in the LST toolbox (www.statistical-modelling.de/lst.html) for SPM (https://www.fil.ion.ucl.ac.uk/spm/). Only T2 FLAIR images were used to detect the WMH lesions. For each individual, T2-FLAIR images were registered to the T1-weighted images using an affine transformation and the T1-weighted images were registered to the MNI brain atlas by non-linear transformation. Then, two transformations were concatenated to transform the T2-FLAIR images and corresponding WMH lesions images into the reference space. ^1^ WMH lesions were divided into PWMH or DWMH according to the distance from the lateral ventricles (>10 mm was considered to be deep WMH). ^2^

***Arterial Flow Simulation***

(1) Cerebral vessel segmentation was completed on MRA images using the previously described methods.^3^ The vessels were then reconstructed into 3D space to allow interactive manual selection of inflow vessel branches, including internal carotid and basilar artery. All segmentation results were manually checked to ensure that at least all secondary branches were completely segmented (M2/P2/A2).

(2) The centerlines of vessels were extracted using the Skeleton 3D toolbox.^4^ The topological connections among vascular branches were organized by evaluating the adjacency of centerline points. The inflow branches were manually identified, while all outflow branches were automatically obtained through the topological connections. (3) Each arterial branch was considered a rigid, uniformly cylindrical structure. The blood flow within the vessels was regarded as incompressible Newtonian fluid, and assumed to be a steady laminar flow. Based on these assumptions, the hemodynamic features of each arterial branch were represented using the Hagen-Poiseuille equation as follow:

$$\Delta P=\frac{8\mu L}{\pi r^{4}}Q$$

$$\Delta P=P_{in}-P_{out}$$

Where $\Delta P$ means pressure drop along each branch and could be calculated by subtracting the inflow pressure $P_{in}$ from outflow pressure $P_{out}$. $Q$ represents blood flow rate, $r$ and $L$ represents radius and length, $\mu$ is the dynamic viscosity of blood which is set to 0.003 Pa·s.

Consequently, there were three hemodynamic parameters that need to be resolved for each arterial branch: the inflow pressure, the outflow pressure, and the internal blood flow rate. To solve for the unknows and close the system of equations, boundary conditions were introduced in addition to the application of the Hagen-Poiseuille equation for each branch.

For inlet boundary condition, the mean arterial pressure (MAP) was served as $P_{in}$ for a virtual vascular branch which was generated based on all the manually identified inflow branches (including the internal carotid artery and the basilar artery).^5^ The radius and length of the virtual branch was 1.1 times of the largest radius and length of the manually identified inflow branches, since the downward bifurcation of the vascular tree conforms to the law of Murray. ^6 7^ The MAP was calculated based on diastolic blood pressure (DBP) and systolic blood pressure (SBP) as follow:

$$MAP=DBP+\frac{1}{3}(SBP-DBP)$$

For outlet boundary condition, a patient-specific structured tree was built for each outflow branch until the diameter of the terminal branches in the structured tree was less than 0.1 mm.^8^ The structural tree was a binary tree that began with each exit branch. The radius of every left-side sub-branch decreased continuously with a coefficient of 0.8978, while the radius of every right-side sub-branch decreased continuously with a coefficient of 0.7238 until the radius of the terminal branches was less than 0.1mm. The $P_{out}$ of the terminal branch was 0.3 N/cm^2^.

For bifurcation, the incoming flow was equal to the outgoing flow and pressure was assuming continuity:

$$Q_{pa}=Q_{ch1}+Q_{ch2}+\ldots Q_{chn}$$

$$P_{out}^{pa}=P_{in}^{ch1}=P_{in}^{ch2}=\ldots=P_{in}^{chn}$$

Where $Q_{fa}$ represents the flow rate of parent branch and $Q_{ch1},\ldots,Q_{chn}$ represents the flow rate of all children branch. $P_{out}^{fa}$ represents the outflow pressure of parent branch and $P_{in}^{ch1},\ldots,P_{in}^{chn}$ represents the inflow pressure of all children branch.

Overall, the three unknown hemodynamic features of each branch in the arterial tree formed a closed system of equations comprising the aforementioned Hagen-Poiseuille equation and corresponding boundary conditions. Since all the governing equations were linear, the flow features for each vascular branch were directly obtained using matrix inversion methods. Based on the pressures at the inflow ($P_{in}$) and outflow ($P_{out}$) of each branch, the mean pressure and pressure drop was calculated. All data processing was performed on MATLAB (version 2022a, MathWorks, Natick, Massachusetts, USA).

***Evaluation Model Performance***

To validate the performance of the simplified hemodynamic model, this study employed Flow-MRI to measure blood flow in critical intracranial vascular branches and compared it with simulation results. Data were derived from three healthy volunteers, with both TOF MRA and Flow-MRI images being concurrently collected. The protocol of Flow-MRI was as follow: TE=3.6ms, TR=8.0ms, flip angle=10°, matrix size=224×224×20, voxel size=0.94mm×0.94mm×2mm, VENC=120cm/s. Flow rate in the left and right internal carotid arteries, middle cerebral arteries, posterior cerebral arteries, and basilar artery, considered as measurement standards, was obtained through the GTFlow tools (www.gyrotools.com). Three different planes were manually selected on each arterial segment, and the mean value was calculated to represent the flow rate of this artery. Subsequent results from simulations using TOF-MRA were compared with those from Flow-MRI.

**Reference**

1. Mouches P, Forkert ND. A statistical atlas of cerebral arteries generated using multi-center MRA datasets from healthy subjects. *Scientific data* 2019;6(1):29.

2. Griffanti L, Jenkinson M, Suri S, et al. Classification and characterization of periventricular and deep white matter hyperintensities on MRI: a study in older adults. *Neuroimage* 2018;170:174-81.

3. Zhang BY, Wang YZ, Wang B, et al. MRI-Based Investigation of Association Between Cerebrovascular Structural Alteration and White Matter Hyperintensity Induced by High Blood Pressure. *Journal of Magnetic Resonance Imaging* 2021;54(5):1516-26. doi: 10.1002/jmri.27815

4. Kollmannsberger P, Kerschnitzki M, Repp F, et al. The small world of osteocytes: connectomics of the lacuno-canalicular network in bone. *New Journal of Physics* 2017;19(7):073019.

5. Zhu J, Teolis S, Biassou N, et al. Tracking the adaptation and compensation processes of patients’ brain arterial network to an evolving glioblastoma. *IEEE transactions on pattern analysis and machine intelligence* 2020;44(1):488-501.

6. Helthuis JH, van Doormaal TP, Amin-Hanjani S, et al. A patient-specific cerebral blood flow model. *Journal of Biomechanics* 2020;98:109445.

7. Murray CD. THE PHYSIOLOGICAL PRINCIPLE OF MINIMUM WORK APPLIED TO THE ANGLE OF BRANCHING OF ARTERIES. *J Gen Physiol* 1926;9(6):835-41. doi: 10.1085/jgp.9.6.835 [published Online First: 1926/07/20]

8. Olufsen MS, Peskin CS, Kim WY, et al. Numerical simulation and experimental validation of blood flow in arteries with structured-tree outflow conditions. *Ann Biomed Eng* 2000;28(11):1281-99. doi: 10.1114/1.1326031 [published Online First: 2001/02/24]

**Figure S1**. Schematic representation of the simplified hemodynamic model implementation.

1. Segmentation; B) center linear extraction; C) simplified hemodynamic model.


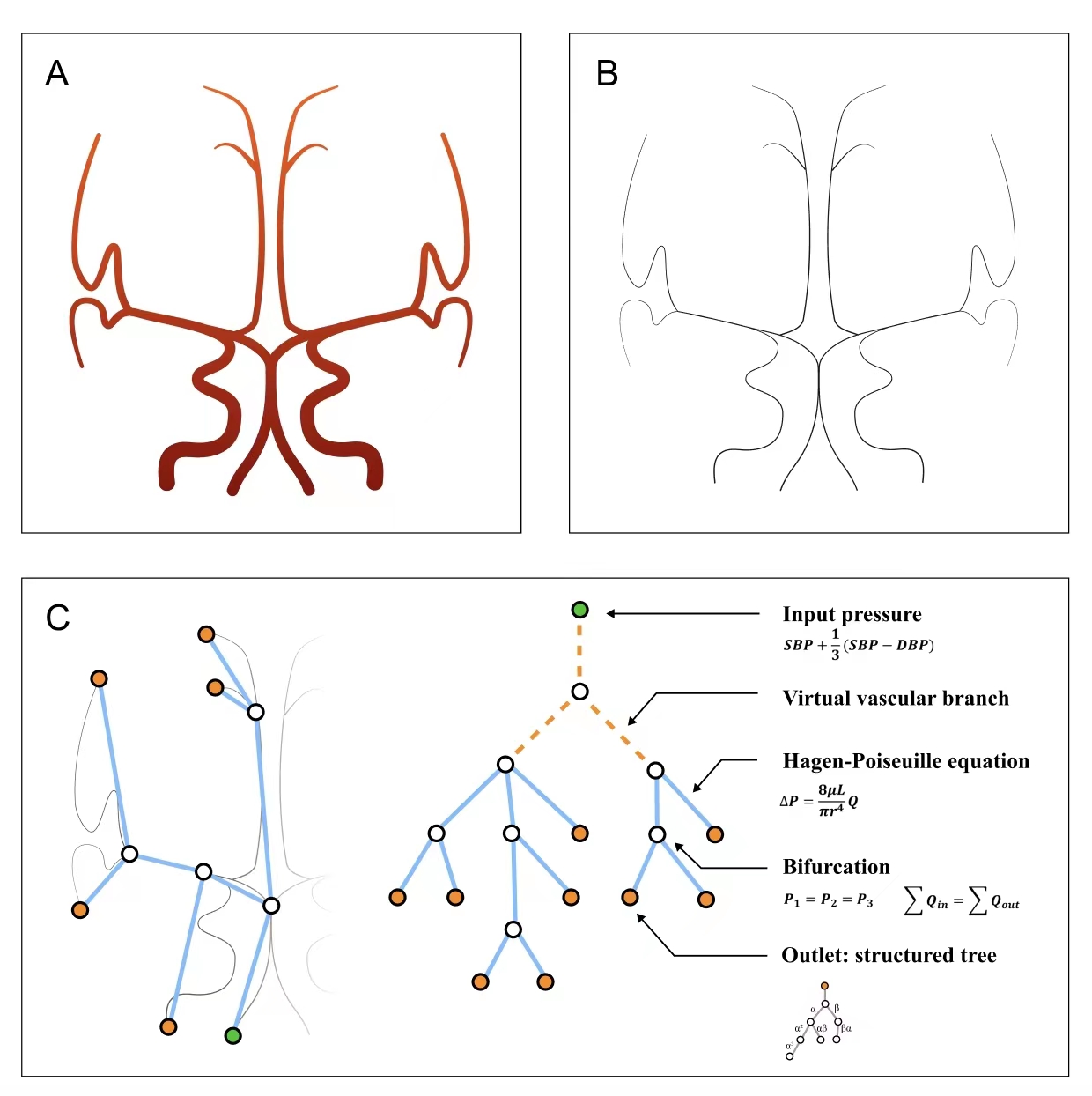


**Figure S2**. Histogram of distance between white matter hyperintensity and the nearest terminal arterial branch.


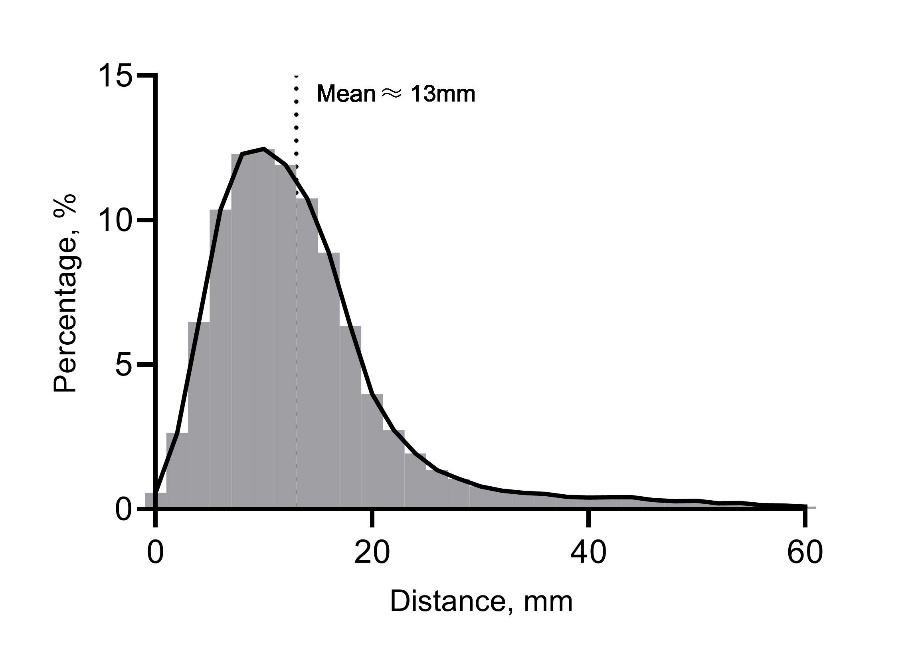


**Figure S3**. Morphology between Adjacent-to-Lesion Terminal Arterial Branches and other branches under different threshold.


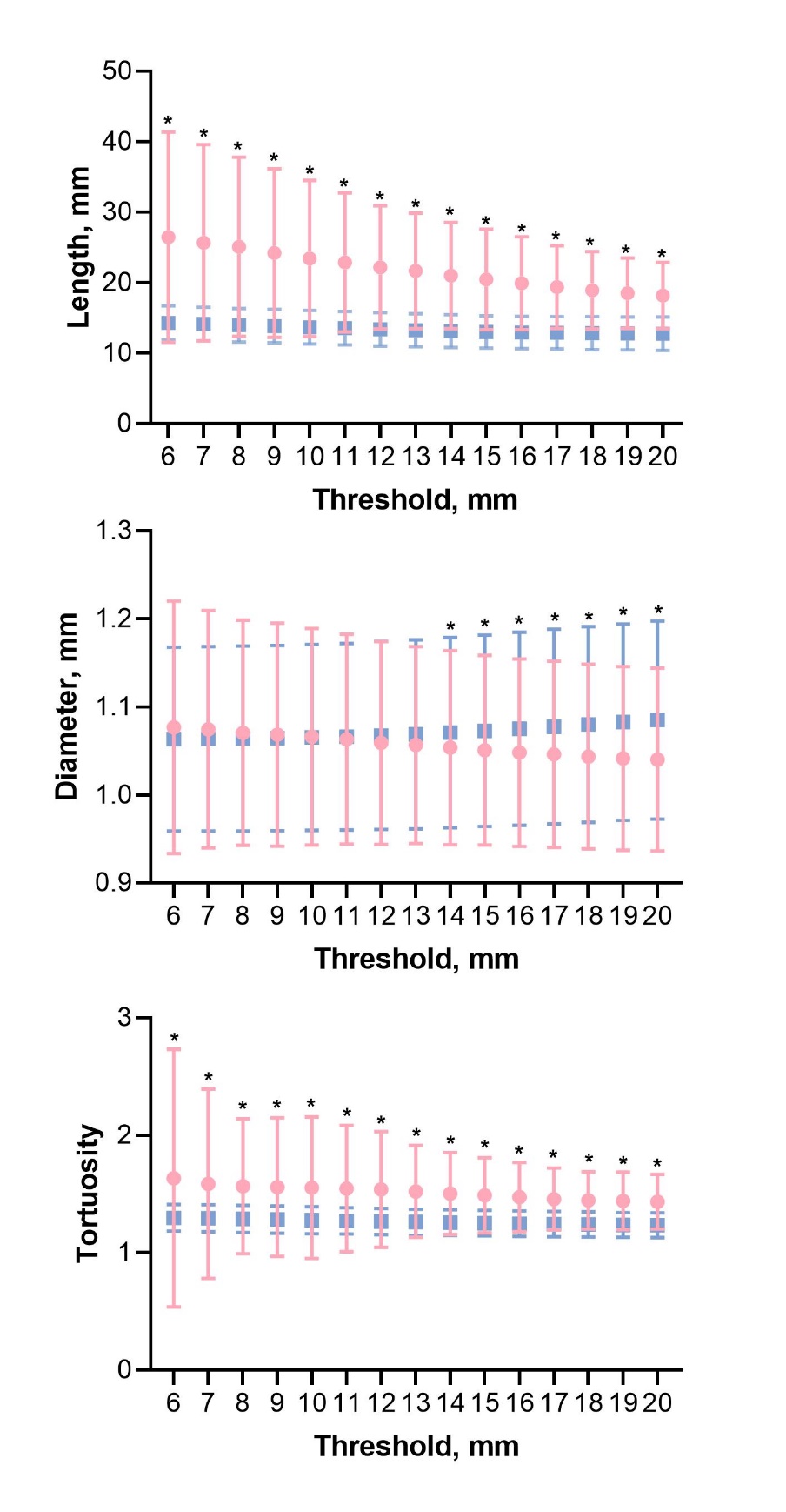


* indicates a significant difference between the two groups, with results corrected using the Bonferroni method. The red section represents Adjacent-to-Lesion Terminal Arterial Branches, while the blue section represents other terminal branches.

**Figure S4**. Flow features between Adjacent-to-Lesion Terminal Arterial Branches and other branches under different threshold.


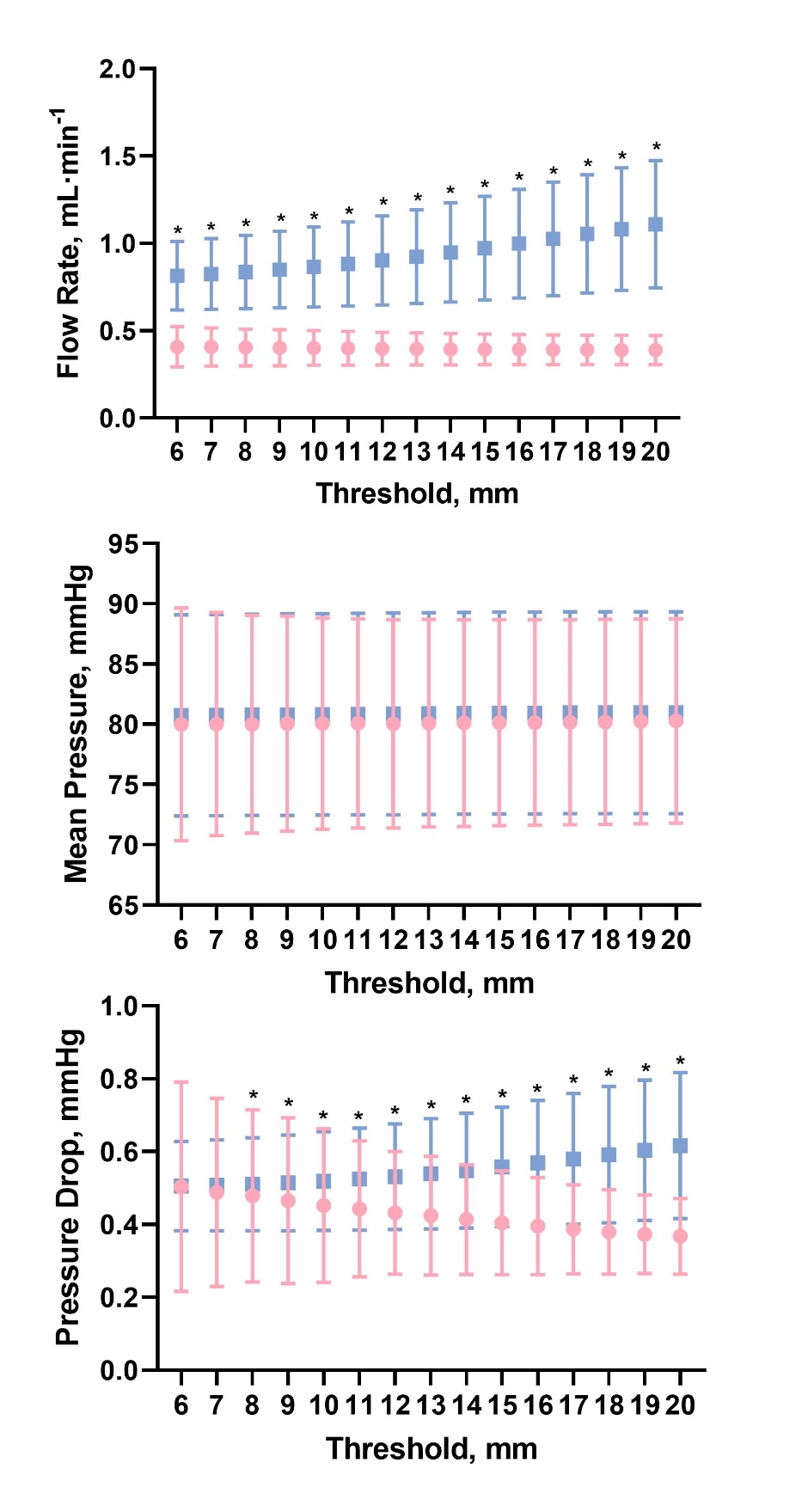


* indicates a significant difference between the two groups, with results corrected using the Bonferroni method. The red section represents Adjacent-to-Lesion Terminal Arterial Branches, while the blue section represents other terminal branches.

**Figure S5**. Cerebral arterial flow rate between Flow-MRI and simulation.

A) Flow rate of each intracranial arterial branch; B) Scatterplot comparison


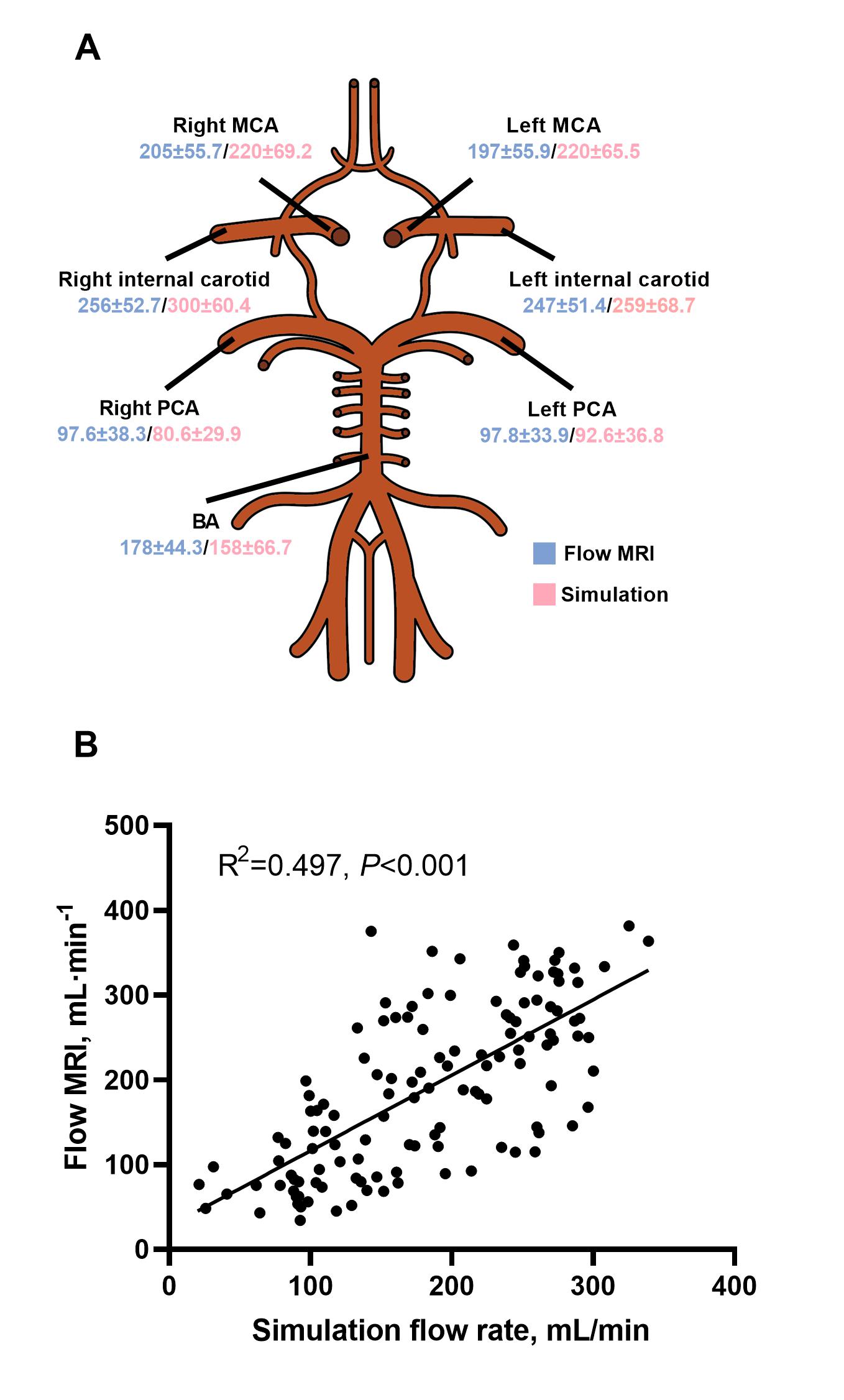


**Table S1**. Association between cerebral arterial flow pattern and white matter hyperintensity in hypertensive individuals.

|  | Bivariable model | | Adjusted Model 1 | | Adjusted Model 2 | |
| --- | --- | --- | --- | --- | --- | --- |
|  | β (95% CI) | P value | β (95% CI) | P value | β (95% CI) | P value |
| ***Total WMHV*** |  |  |  |  |  |  |
| Mean flow rate, mL/min | 0.03 (-0.03 to 0.08) | 0.36 | 0.07 (0.03 to 0.12) | 0.001 | 0.08 (0.03 to 0.12) | 0.001 |
| Mean pressure, mmHg | 0.02 (0.008 to 0.03) | 0.001 | 0.02 (0.02 to 0.03) | p<0.001 | 0.03 (0.02 to 0.03) | p<0.001 |
| Mean pressure drop, mmHg | -0.27 (-1.13 to 0.59) | 0.54 | -0.11 (-0.84 to 0.61) | 0.76 | -0.18 (-0.91 to 0.54) | 0.62 |
| ***PWMHV*** |  |  |  |  |  |  |
| Mean flow rate, mL/min | 0.01 (-0.05 to 0.07) | 0.7 | 0.06 (0.01 to 0.11) | 0.02 | 0.07 (0.01 to 0.12) | 0.01 |
| Mean pressure, mmHg | 0.02 (0.007 to 0.03) | 0.002 | 0.03 (0.02 to 0.04) | p<0.001 | 0.03 (0.02 to 0.04) | p<0.001 |
| Mean pressure drop, mmHg | -0.20 (-1.16 to 0.76) | 0.69 | -0.06 (-0.89 to 0.77) | 0.88 | -0.13 (-0.96 to 0.70) | 0.76 |
| ***DWMHV*** |  |  |  |  |  |  |
| Mean flow rate, mL/min | -0.03 (-0.10 to 0.04) | 0.35 | 0.01 (-0.05 to 0.08) | 0.68 | 0.01 (-0.05 to 0.08) | 0.68 |
| Mean pressure, mmHg | 0.008(-0.006 to 0.02) | 0.26 | 0.02 (0.003 to 0.03) | 0.02 | 0.02 (0.003 to 0.03) | 0.02 |
| Mean pressure drop, mmHg | 0.30 (-0.78 to 1.38) | 0.59 | 0.64 (-0.36 to 1.65) | 0.21 | 0.53 (-0.48 to 1.54) | 0.30 |

Model 1 adjusted for age and sex; Model 2 adjusted for age, sex and vascular risk factors including cigarette use, alcohol consumption, diabetes, hyperlipidaemia and history of cardiovascular disease. DWMHV, deep white matter hyperintensity lesion volume; PWMHV, periventricular white matter hyperintensity lesion volume; and WMHV, white matter hyperintensity lesion volume.

**Table S2**. Association between cerebral arterial flow pattern and white matter hyperintensity in non-hypertensive individuals.

|  | Bivariable model | | Adjusted Model 1 | | Adjusted Model 2 | |
| --- | --- | --- | --- | --- | --- | --- |
|  | β (95% CI) | P value | β (95% CI) | P value | β (95% CI) | P value |
| ***Total WMHV*** |  |  |  |  |  |  |
| Mean flow rate, mL/min | 0.21 (0.10 to 0.32) | p<0.001 | 0.12 (0.05 to 0.20) | 0.001 | 0.12 (0.05 to 0.20) | 0.001 |
| Mean pressure, mmHg | 0.07 (0.05 to 0.09) | p<0.001 | 0.03 (0.02 to 0.05) | p<0.001 | 0.03 (0.02 to 0.05) | p<0.001 |
| Mean pressure drop, mmHg | 4.17 (2.35 to 5.99) | p<0.001 | 1.4 (0.11 to 2.69) | 0.03 | 1.25 (-0.04 to 2.54) | 0.06 |
| ***PWMHV*** |  |  |  |  |  |  |
| Mean flow rate, mL/min | 0.21 (0.09 to 0.33) | p<0.001 | 0.12 (0.04 to 0.20) | 0.005 | 0.12 (0.04 to 0.20) | 0.005 |
| Mean pressure, mmHg | 0.07 (0.05 to 0.09) | p<0.001 | 0.04 (0.02 to 0.05) | p<0.001 | 0.04 (0.02 to 0.05) | p<0.001 |
| Mean pressure drop, mmHg | 4.54 (2.58 to 6.5) | p<0.001 | 1.59 (0.15 to 3.03) | 0.03 | 1.44 (-0.005 to 2.88) | 0.051 |
| ***DWMHV*** |  |  |  |  |  |  |
| Mean flow rate, mL/min | 0.15 (0.01 to 0.29) | 0.03 | 0.07 (-0.05 to 0.18) | 0.27 | 0.06 (-0.06 to 0.18) | 0.34 |
| Mean pressure, mmHg | 0.06 (0.03 to 0.09) | p<0.001 | 0.03 (0.007 to 0.05) | 0.01 | 0.03 (0.005 to 0.05) | 0.02 |
| Mean pressure drop, mmHg | 4.44 (2.06 to 6.82) | p<0.001 | 1.83 (-0.20 to 3.86) | 0.08 | 1.67 (-0.37 to 3.71) | 0.11 |

Model 1 adjusted for age and sex; Model 2 adjusted for age, sex and vascular risk factors including cigarette use, alcohol consumption, diabetes, hyperlipidaemia and history of cardiovascular disease. DWMHV, deep white matter hyperintensity lesion volume; PWMHV, periventricular white matter hyperintensity lesion volume; and WMHV, white matter hyperintensity lesion volume.

**Table S3**. Association between cerebral arterial flow pattern and white matter hyperintensity in subgroups.

|  | Mean flow rate and WMH | | Mean pressure and WMH | | Mean pressure drop and WMH | |
| --- | --- | --- | --- | --- | --- | --- |
|  | β (95% CI) | P value | β (95% CI) | P value | β (95% CI) | P value |
| All (N=2631) | **0.10 (0.06 to 0.14)** | **p<0.001** | **0.03 (0.02 to 0.04)** | **p<0.001** | 0.40 (-0.24 to 1.04) | 0.22 |
| Sample 1 (N=263) | **0.19 (0.06 to 0.32)** | **0.004** | 0.02 (-0.001 to 0.05) | 0.06 | 0.06 (-2.31 to 2.43) | 0.96 |
| Sample 2 (N=263) | 0.03 (-0.08 to 0.15) | 0.55 | **0.02 (0.003 to 0.05)** | **0.03** | -1.3 (-3.26 to 0.65) | 0.19 |
| Sample 3 (N=263) | 0.05 (-0.08 to 0.17) | 0.45 | 0.02 (-0.002 to 0.05) | 0.07 | -0.88 (-2.74 to 0.99) | 0.36 |
| Sample 4 (N=263) | **0.17 (0.02 to 0.31)** | **0.02** | **0.06 (0.03 to 0.09)** | **p<0.001** | **2.69 (0.23 to 5.15)** | **0.03** |
| Sample 5 (N=263) | **0.13 (0.01 to 0.26)** | **0.03** | **0.05 (0.03 to 0.08)** | **p<0.001** | 1.18 (-0.75 to 3.1) | 0.23 |
| Sample 6 (N=263) | **0.21 (0.09 to 0.33)** | **0.001** | **0.04 (0.02 to 0.06)** | **0.001** | 1.5 (-0.34 to 3.34) | 0.11 |
| Sample 7 (N=263) | **0.22 (0.10 to 0.34)** | **0.001** | **0.04 (0.010 to 0.06)** | **0.007** | 0.24 (-1.85 to 2.33) | 0.82 |
| Sample 8 (N=263) | 0.09 (-0.03 to 0.20) | 0.13 | **0.03 (0.005 to 0.05)** | **0.02** | -0.16 (-2.14 to 1.83) | 0.88 |
| Sample 9 (N=263) | **0.16 (0.05 to 0.27)** | **0.005** | **0.04 (0.01 to 0.06)** | **0.001** | 0.64 (-1.43 to 2.7) | 0.55 |
| Sample 10 (N=263) | **0.22 (0.11 to 0.33)** | **p<0.001** | **0.04 (0.02 to 0.06)** | **0.001** | 0.43 (-1.41 to 2.28) | 0.64 |

All models were adjusted for age, sex and vascular risk factors including cigarette use, alcohol consumption, diabetes, hyperlipidemia, hypertension and history of cardiovascular disease. WMH, white matter hyperintensity.

**Table S4**. Association between flow features of Adjacent-to-Lesion Terminal Arterial Branches and white matter hyperintensity in subgroups.

|  | ALTAB flow rate and WMH | | ALTAB pressure and WMH | | ALTAB pressure drop and WMH | |
| --- | --- | --- | --- | --- | --- | --- |
|  | β (95% CI) | P value | β (95% CI) | P value | β (95% CI) | P value |
| All (N=2631) | **2.67 (2.1 to 3.25)** | **p<0.001** | **0.04 (0.04 to 0.05)** | **p<0.001** | **-0.86 (-1.19 to -0.53)** | **p<0.001** |
| Sample 1 (N=263) | **4.33 (0.37 to 0.35)** | **p<0.001** | **0.06 (0.40 to 0.38)** | **p<0.001** | -0.88 (0.33 to 0.31) | 0.12 |
| Sample 2 (N=263) | **3.67 (0.53 to 0.51)** | **p<0.001** | **0.04 (0.53 to 0.51)** | **p<0.001** | **-1.36 (0.50 to 0.48)** | **0.004** |
| Sample 3 (N=263) | 0.16 (0.42 to 0.40) | 0.87 | 0.008 (0.42 to 0.40) | 0.5 | **-1.97 (0.47 to 0.45)** | **p<0.001** |
| Sample 4 (N=263) | **2.85 (0.46 to 0.44)** | **0.005** | **0.05 (0.48 to 0.46)** | **p<0.001** | -0.38 (0.44 to 0.42) | 0.48 |
| Sample 5 (N=263) | **3.11 (0.40 to 0.38)** | **0.002** | **0.05 (0.41 to 0.39)** | **p<0.001** | -0.78 (0.38 to 0.36) | 0.16 |
| Sample 6 (N=263) | 1.52 (0.43 to 0.41) | 0.1 | **0.04 (0.46 to 0.44)** | **p<0.001** | 0.26 (0.42 to 0.40) | 0.67 |
| Sample 7 (N=263) | **1.69 (0.42 to 0.40)** | **0.043** | **0.02 (0.42 to 0.40)** | **0.03** | **-1.26 (0.44 to 0.42)** | **0.001** |
| Sample 8 (N=263) | **3.51 (0.43 to 0.41)** | **p<0.001** | **0.04 (0.43 to 0.40)** | **p<0.001** | -0.86 (0.40 to 0.38) | 0.1 |
| Sample 9 (N=263) | **2.67 (0.46 to 0.44)** | **0.006** | **0.04 (0.48 to 0.46)** | **p<0.001** | **-1.37 (0.46 to 0.44)** | **0.02** |
| Sample 10 (N=263) | **3.19 (0.41 to 0.39)** | **0.001** | **0.05 (0.44 to 0.42)** | **p<0.001** | -0.87 (0.39 to 0.37) | 0.07 |

All models were adjusted for age, sex and vascular risk factors including cigarette use, alcohol consumption, diabetes, hyperlipidemia, hypertension and history of cardiovascular disease. WMH, white matter hyperintensity. ALTAB, Adjacent-to-Lesion Terminal Arterial Branch.

**Table S5**. Two sample t-test between Adjacent-to-Lesion Terminal Arterial Branches and other arterial branches in subgroups.

|  | Length | | Radius | | Tortuosity | | Flow rate | | Mean pressure | | Pressure drop | |
| --- | --- | --- | --- | --- | --- | --- | --- | --- | --- | --- | --- | --- |
|  | T | P | T | P | T | P | T | P | T | P | T | P |
| All (N=2631) | 50.6 | p<0.001 | -2.38 | 0.02 | 32.9 | p<0.001 | -94.2 | p<0.001 | -2.22 | 0.03 | -26.3 | p<0.001 |
| Sample 1 (N=263) | 16.4 | p<0.001 | -0.52 | 0.6 | 9.1 | p<0.001 | -32.2 | p<0.001 | -0.82 | 0.41 | -8.8 | p<0.001 |
| Sample 2 (N=263) | 16.5 | p<0.001 | -1.52 | 0.13 | 12.6 | p<0.001 | -31.4 | p<0.001 | -0.70 | 0.48 | -8.2 | p<0.001 |
| Sample 3 (N=263) | 15.4 | p<0.001 | -1.86 | 0.06 | 8.9 | p<0.001 | -29.5 | p<0.001 | -0.79 | 0.43 | -9.3 | p<0.001 |
| Sample 4 (N=263) | 15.9 | p<0.001 | -0.73 | 0.47 | 11.1 | p<0.001 | -29.2 | p<0.001 | -1.85 | 0.06 | -9.8 | p<0.001 |
| Sample 5 (N=263) | 15.2 | p<0.001 | -0.90 | 0.37 | 10.7 | p<0.001 | -30.6 | p<0.001 | -0.82 | 0.41 | -8.1 | p<0.001 |
| Sample 6 (N=263) | 15.2 | p<0.001 | -1.10 | 0.27 | 14.3 | p<0.001 | -30.3 | p<0.001 | -1.62 | 0.11 | -11.2 | p<0.001 |
| Sample 7 (N=263) | 18.4 | p<0.001 | -1.13 | 0.26 | 9.9 | p<0.001 | -31.1 | p<0.001 | -0.71 | 0.48 | -8.3 | p<0.001 |
| Sample 8 (N=263) | 15.6 | p<0.001 | -2.66 | 0.008 | 9.5 | p<0.001 | -28.8 | p<0.001 | -0.98 | 0.33 | -6.6 | p<0.001 |
| Sample 9 (N=263) | 15.4 | p<0.001 | -1.19 | 0.23 | 10.9 | p<0.001 | -30.7 | p<0.001 | -1.07 | 0.28 | -7.2 | p<0.001 |
| Sample 10 (N=263) | 17.1 | p<0.001 | -1.42 | 0.16 | 10.0 | p<0.001 | -30.0 | p<0.001 | -0.90 | 0.37 | -8.3 | p<0.001 |

T represents the T-statistic. In this table, a positive value indicates that ALTAB > other branches, while a negative value indicates that ALTAB < other branches. ALTAB, Adjacent-to-Lesion Terminal Arterial Branch.
